# Airway clearance physiotherapy and health-related quality of life in cystic fibrosis

**DOI:** 10.1371/journal.pone.0276310

**Published:** 2022-10-18

**Authors:** Sandra Gursli, Alexandra Quittner, Reidun Birgitta Jahnsen, Bjørn Skrede, Britt Stuge, Egil Bakkeheim

**Affiliations:** 1 National Resource Centre for Cystic Fibrosis, Oslo University Hospital, Oslo, Norway; 2 Miami Children’s Research Institute, Miami, Florida, United States of America; 3 Department of Neurosciences for Children, Oslo University Hospital, Oslo, Norway; 4 Institute of Health and Society, CHARM, University of Oslo, Oslo, Norway; 5 Department of Pulmonary Medicine, Oslo University Hospital, Oslo, Norway; 6 Division of Orthopaedic Surgery, Department of Research and Development, Oslo University Hospital, Oslo, Norway; IRCCS E. Medea, ITALY

## Abstract

**Objective:**

Airway clearance physiotherapy is recommended in cystic fibrosis, but limited evidence exists to suggest how much treatment is enough. As a secondary analysis of a prior study investigating the safety, efficacy, and participants’ perceptions of a novel airway clearance technique, specific cough technique (SCT) compared to forced expiration technique (FET), we aimed to evaluate whether the intervention was associated with changes in health-related quality of life (HRQoL).

**Methods:**

We conducted randomised, controlled individual trials with six adults (N-of-1 RCTs). Each trial included eight weeks of treatment, twice a week, using saline inhalation in horizontal positions, one with SCT and one with FET, in random order. Efficacy was measured by sputum wet weight (g) after each session. Perceived usefulness and preference were self-reported at the end of the study. Lung function was assessed at baseline and at the end of study. HRQoL was measured using the Cystic Fibrosis Questionnaire-Revised (CFQ-R) at baseline (week 1) and at completion of the study (week 8). Individual HRQoL scores (0–100) were coded and analysed using CFQ-R Software Program, version 2.0.

**Results:**

Patient-reported outcomes were completed by all subjects. Individual CFQ-R-Respiratory Symptoms Scores (CFQ-R-RSS) showed a positive change, meeting the minimal important difference (MID) ≥ 4 points in five participants and a negative change in one individual. A strong correlation (r = 0.94 (p<0.01) was found between total sputum weight (g) and the positive changes in CFQ-R-RSS, and between changes in lung function and CFQ-R-RSS (r = 0.84 (p = 0.04).

**Conclusion:**

The airway clearance intervention was associated with clinically meaningful changes in patient-reported symptoms on the CFQ-R in the majority of the participants. This finding warrants further investigation regarding treatment, duration and frequency. A long-term study may reveal beneficial effects on other clinically meaningful endpoints, such as pulmonary exacerbations, high-resolution computed tomography scores and HRQoL.

**Trial registration:**

The study was registered in ClinicalTrials.gov, under the number NCT0 1266473.

## Introduction

Lung disease in cystic fibrosis (CF) is characterized by increased mucus production and reduced mucus clearance, leading to a process of inflammation, infection, structural changes, and potentially reduced participation in everyday activities and quality of life [1–3]. Airway clearance is a fundamental part of the treatment regimen for CF [4], and effective mucus clearance is essential to reduce symptoms and optimize treatment [1]. It is also considered as one of the most time-consuming, burdensome aspect of the CF treatment regimen [5].

Airway Clearance Physiotherapy (ACP) comprises use of different airway clearance techniques (ACTs) [6] interacting to mobilise and remove sputum. Several studies have investigated the effectiveness of ACTs using different outcome measures. Currently, there is a lack of evidence supporting the use of one particular airway clearance technique (ACT) [7].

In a previous study we performed a series of N-of-1 randomized, controlled trials and have reported on the individual efficacy, safety and participants’ perceptions of a novel ACT–Specific Cough Technique (SCT) compared to Forced Expiration Technique (FET) [8].

As individuals with CF live longer due to better care and treatment, HRQoL has been commonly studied [3] to assess aspects of daily functioning related to health. HRQoL has been shown to be negatively affected by treatment burden [9], frequency of pulmonary exacerbations [4], complications, isolation, treatment complexity and comorbidities, such as CF-related diabetes, and depression and anxiety [10]. HRQoL has become an important outcome measure in clinical trials and evaluation of disease management primarily because these measures reflect the patient’s own experience and perceptions of functioning [11]. Therefore, it is critical to assess the impact of new treatments or changes in the treatment regimen on individual, daily functioning [10]. Further, The US Food and Drug Administration mandates that new treatments include patient-reported outcome measures, such as the CFQ-R [3, 12].

Disease-specific instruments are needed to evaluate the effectiveness of interventions [13, 14] and the benefits of existing or new treatments [12]. The CFQ-R is the “gold standard” quality of life measure for CF, and the most widely used instrument in CF-related research. The CFQ-R includes several symptoms scales, most notably, respiratory symptoms, as well as physical, social and emotional functioning, and treatment burden [15]. The CFQ-R has demonstrated strong psychometric properties (several forms of reliability and validity, and responsiveness) [16].

ACP focuses on reducing respiratory symptoms, pulmonary infections, and preventing loss of function and health. Thus, ACP has the potential to positively impact on HRQoL. The CFQ-R can be used in research or clinical practice to measure the effects of ACP interventions [17]. However, to date few studies have investigated the relationship between ACP and HRQoL [7]. There is a lack of data demonstrating the impact of airway clearance interventions on HRQoL and no clear evidence for its optimal duration and frequency [3, 18].

The objective of the present secondary analysis of the prior study [8] was to assess the effectiveness of the intervention, by evaluating whether participation in the trial led to changes in HRQoL and in particular, clinical meaningful changes in CFQ-R-Respiratory Symptoms Scores (CFQ-R-RSS). A second aim was to examine potential associations between HRQoL and other clinical outcomes, such as sputum production and pulmonary function.

## Materials and methods

### Study design

We conducted a pre-planned secondary analysis to a series of randomized controlled, individual trials (N-of-1 RCT) in adults with CF using a multiple crossover sequence in eight pairs of treatment periods [8]. The rationale for using the N-of-1 approach and the participant flow is described previously [8].

Each participant performed treatment in pairs, serving as their own control with two interventions in each pair, one with SCT and one with FET in randomized order. Thus, performing 16 treatments during eight treatment periods. The treatment pairs were performed on two consecutive days separated by 24 hours. Perceived usefulness and preference were self-reported at the end of study. Further details were reported previously [8]. HRQoL was self-reported at baseline and end of trial using the Cystic Fibrosis Questionnaire Revised (CFQ-R). Lung function was measured at baseline and end of study.

CFQ-R

### Working model and intervention

The present trial was designed to cover defined quality criteria for treatment and learning: Gentle, Efficient, Motivating, and Self-supporting (GEMS) [19, 20]. Target group was identified with a need for airway clearance therapy, according to inclusion criteria. The treatment method is based on physiological principles of ventilation and deposition and on optimised treatments to mobilise and remove sputum according to the LMR–principle: Loosen–Move–Remove [20]. The method for both treatment and learning represents a dynamic process [20].

The intervention included use of a nebulized bronchodilator followed by either nebulized hypertonic or isotonic saline, depending on the study subject’s routine treatments.

The interactive value of using mucoactive therapy as part of ACP probably increases efficacy and improves airway clearance since the therapies work together to optimize outcomes. Participants used nebulised saline during treatment in side-lying horizontal positions to facilitate mobilization of mucus to larger airways alternating with sputum removal using either FET or SCT in random order [8]. The breathing pattern used during nebulisation was adapted to the individual, using a standardised protocol to address ventilation and deposition The intervention protocol is previously thoroughly described [8].

**Fig 1:**
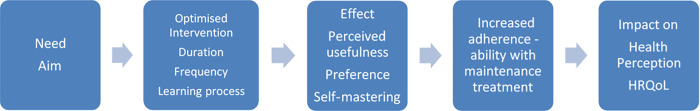
Treatment method.

Importantly, perceived efficacy and usefulness with treatment is a prerequisite for expecting adherence with maintenance treatment and thus impact on perceived health and HRQoL in a positive direction. The treatment method is visualised as a presupposition for expecting adherence with treatment and impact on HRQoL (Fig 1).

### Study subjects

Participants were recruited consecutively from the patient directory at the regional Oslo CF Centre. The CF diagnosis of the participants was based on clinical symptoms, sweat chloride levels > 59 mmol/L and two CF mutations in Cystic fibrosis transmembrane conductance regulator. Inclusion criteria were: CF diagnosis, age ≥ 18 years, sputum expectorated more than five ml in a treatment session, either isotonic or hypertonic saline inhalation as part of treatment, or dornase alpha. Exclusion criteria were: respiratory failure, active haemoptysis, pregnancy and acquisition with multiresistant Pseudomonas aeruginosa, Burkholderia cepacia, atypical Mycobacteria or Methicillin-Resistant Staphylococcus aureus. All participants were clinically stable at the time of entry, i.e., required no treatment for a pulmonary exacerbation within past two weeks. None were listed for transplant evaluation. The study was performed from August 2010 until April 2011 at the location and premises of the Department of Lung Medicine at Oslo University Hospital. The Regional Ethics Committee in Oslo approved this study (2010/802). All participants provided written informed consent. The study was registered in ClinicalTrials.gov, under the number NCT0 1266473.

### Outcome measures

In the previously reported clinical trial [8] sputum weight was the primary outcome whereas Health Related Quality of life (HRQoL) was listed as one of four outcomes in the analysis plan. In this present secondary analysis of a prior study [8], we decided post hoc, based on the clinical relevance of HRQoL, to use HRQoL as primary outcome and to explore the associations between HRQoL and sputum weight and lung function.

### Primary outcome: HRQoL

HRQoL was measured at baseline (week 1) and completion of the study (week 8) using the CFQ-R for adolescents and adults (CFQ-R 14 +). The CFQ-R was developed to assess disease-specific HRQoL domains. CFQ-R consists of 49 items on 12 domains: 9 quality-of-life domains, an overall Health Perceptions Scale and three symptom scales (weight, respiratory, digestion). The instrument is validated in Denmark (2008), Sweden (2017) and translated into Norwegian (National Resource Centre for CF, 2009).

The general domains encompass Physical Functioning, Vitality, Health Perceptions, Emotional, Social and Role Functioning. CF specific domains encompass Body Image, Eating Disturbances, Treatment Burden, and Respiratory and Digestive Symptoms [16]. Patients self-reported on a scale (0–100), with a higher score indicating better HRQoL. The symptoms scales have been developed to assess changes in symptoms over the past 2 weeks [12, 16]. The CFQ-R Respiratory Symptoms domain has been approved by U.S. Food and Drug Administration for use as an endpoint in clinical trials [12, 16]. The minimal important difference (MID) for change is ≥ 4 points, determined at the individual level [21]. The CFQ-R was chosen to examine potential changes in HRQoL, in particular the Respiratory Symptoms domain.

#### Secondary outcomes: The association between HRQoL and lung function and sputum weight respectively

*Lung function*. Spirometry was performed by blinded assessor using a SensorMedicsVMax 20c (Sensor Medics Diagnostics, Yorba Linda, CA, USA). Lung function was assessed at baseline and at the end of the study using forced expiratory flow volumes according to the European Respiratory Society/American Thoracic Society guidelines using reference values from Knudson et.al (1983) [22]. Forced vital capacity (FVC), forced expiratory volume in 1 s (FEV1) and forced expiratory flow at 50% remaining FVC (FEF50) and FEF 25–75, were recorded as percent predicted for sex and height.

*Sputum weight*. Sputum was collected throughout each treatment using pre-weighed transparent and gradated specimen tubes (Careiner bio-one, 50 mL, Germany), one for each side-lying horizontal position, which was changed at the halfway point of the intervention. Sputum was weighed wet (g) after each session by a blinded assessor, using a Mettler TOLEDO weighing Balance (EL 202, accuracy: 0.01 g). The balance was calibrated before each measurement and sputum weight was derived from the total weight minus the pre-use tube weight.

### Data analysis

The original protocol was based on a sample size of 10 participants and 10 interventions. However, a recalculation by the statistican before study start found that six participants and 16 interventions gave a sufficient and more feasible sample size [8]. In the present study individual HRQoL scores were scored and analyzed before and after the study, i.e., not separate for the techniques, using CFQ-R Software Program, version 2.0. Pearson’s correlation coefficients were calculated. Linear regression was used to examine the associations between HRQoL and lung function and sputum weight, respectively.

The data were checked and found to be normally distributed. Thus, linear regression was used. Multiple linear regression analyses using a backward elimination procedure was performed with both sputum weight and lung function in the model to examine which outcome was most strongly associated with HRQoL scores [23]. Analyses were performed using Statistical Package for Social Sciences version 25 (SPSS inc. Chicago, Ill). All p-values (two-sided) equal to or below 0.05 were considered significant.

## Results

Six participants (4 males) with CF were recruited. Median age was 35.3 years and they had a median BMI of 23.0 kg/m^2^. Three had a chronic infection with Pseudomonas aeruginosa and three with Staphylococcus aureus. Median FEV1% predicted, (min-max) was 74.0 (29.0–95.0) median FEF 50% predicted, (min-max) was 49.0 (9.0–98.0) and median FEF25–75% predicted (min–max) was 40.5 (10.0–86.0). Further details are provided in a previous publication [8].

Before study entry, all participants used their usual airway clearance therapy as reported at baseline. The majority of the participants used bronchodilators and saline inhalation (5 of 6) as part of the treatment and physical activity/exercise (3 of 6). One used DNase, and autogenic drainage when feeling tight. They were asked to continue with their usual self-administered treatment at home and not to perform treatment prior to interventions. During the study, all participants used bronchodilators, two inhaled isotonic saline and four inhaled hypertonic saline. Four participants used DNAse (Pulmozyme®) and two participants had regular treatment with inhaled antibiotics.

### Health related quality of life (HRQoL)

Participant-reported outcomes were completed by all study subjects. The data were analyzed for change from baseline. Individual CFQ-R-Respiratory Symptoms Scores (0–100), showed a positive change with an MID ≥ 4 points in 5 of 6 participants (+ 22.2, +22.1, +5.6, +5.6, +16.7). (Table 2). A negative change was seen in one participant (-5). Individual CFQ-R Health Perception Scores showed a positive change in all participants (+11.1, +22.3, +22.2, +11.1, +11.1, +22.2). (Table 1).

**Table 1:** Self-reported HRQoL (CFQ-R) at baseline (week 1) and end of study (week 8).

	Participant 1		Participant 2		Participant 3		Participant 4		Participant 5		Participant 6	
Week	1	8	1	8	1	8	1	8	1	8	1	8
Health Perceptions	55.5	66.6	66.6	88.9	44.4	66.6	44.4	55.5	33.3	44.4	44.4	66.6
		+11.1		+22.3		+22.2		+11.1		+11.1		+22.2
Respiratory Symptoms	55.5	77.7	55.5	66.6	44.4	50.0	55.5	61.1	66.6	61.6	55.5	72.2
		+22.2		+11.1		+ 5.6		+5.6		-5.0		+16.7

Only Respiratory Symptoms and Health Perception scores are shown.

Domains that showed a positive change, no change or a negative change in individual participants were Weight (3/3/0), Role Functioning (2/3/1), Vitality (4/1/1), and Eating (0/5/1). The treatment burden domain showed a positive change in three participants and negative in two (3/1/2). Domains that showed a negative individual change were Physical Functioning (1/1/4), one due to another illness in week 7, Social Functioning (0/1/5), and Body Image (0/2/4). The results regarding HRQoL are presented in full detail in S1 Table.

### Lung function and sputum weight

Lung function data from before and after each intervention in week 2 and sputum production data were reported on previously [8]. To be able to assess associations between the primary and secondary outcomes, we recalculated the data on sputum production (g) and added data on lung function measurements from before and after the study. Data were analyzed as change in absolute values and percent predicted from baseline. Table 2 presents the change in FEV1, FEF50, FEF25-75 (percent predicted) from the start to the end of the study (delta) and sputum weight between weeks 1–8 for each individual patient.

**Table 2:** Change (delta) in lung function and total sputum weight from Week 1 and to the end of the study in Week 8.

	Delta FEV_1_	Delta FEF50	Delta FEF25-75	Sputum weight
Participant 1	+10%	+11%	+7%	756 g
Participant 2	+2%	+3%	+3%	489 g
Participant 3	+5%	+4%	-1%	364 g
Participant 4	+5%	+6%	+4%	401 g
Participant 5	-7%	-13%	-9%	268 g
Participant 6	+6%	+2%	0	501g

### Sputum weight and HRQoL

**Fig 2:**
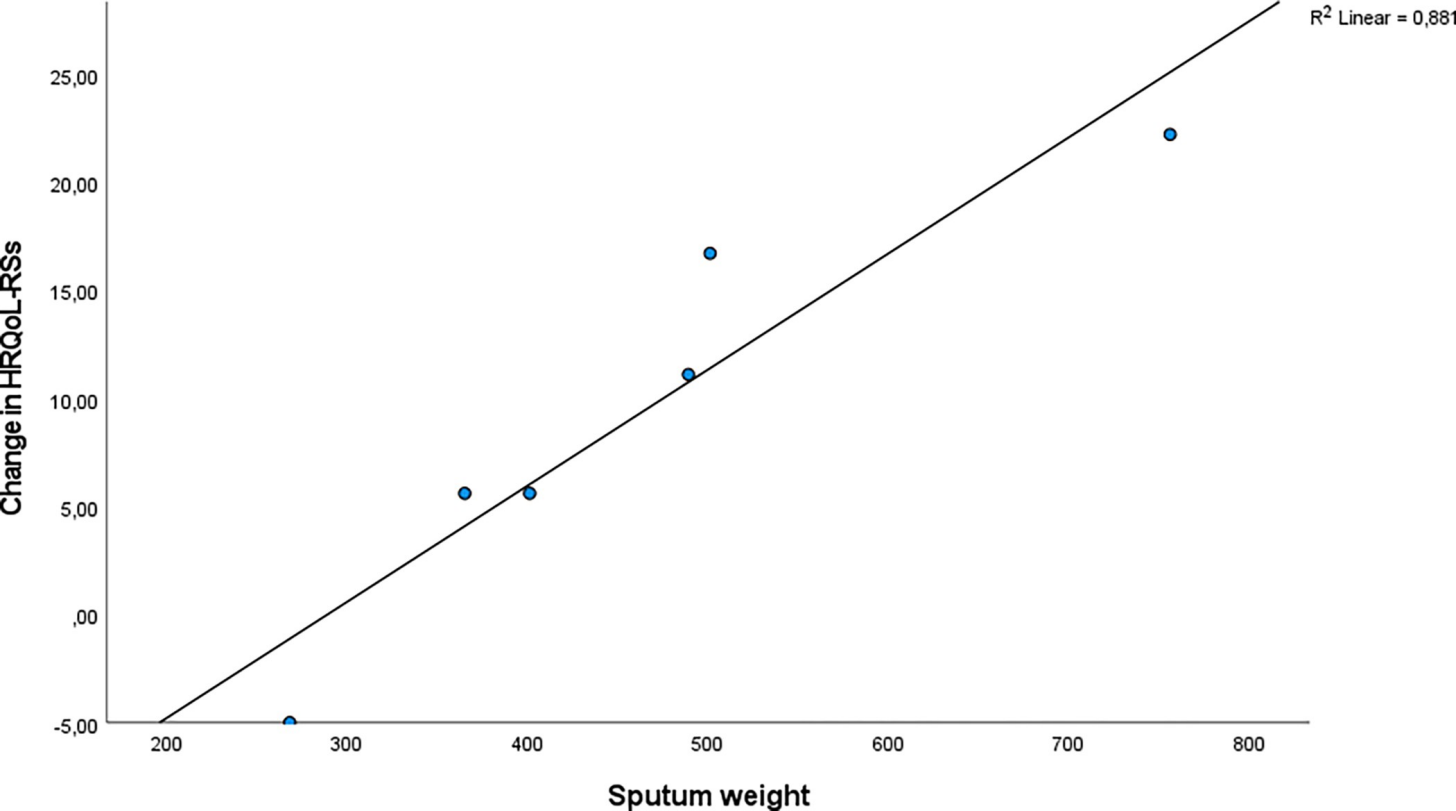
The correlation between total sputum weight (g) produced by the participants during the study (week 1–8) and the change in CFQ-R-RSS.

There was a strong and positive correlation between total sputum weight (g) produced by the participants during the study and positive changes in CFQ-R-Respiratory Symptoms scores (CFQ-R-RSS) r = 0.94 (p<0.01, R^2^ = 0.881 (Fig 2).

There was no significant correlation between sputum weight and the other CFQ-R domains.

In a linear regression model with CFQ-R-RSS as dependent variable, the effect size of 10 grams sputum weight was Beta (CI) 0.54 (0.26, 0.81) (p = 0.006), implying that 10 grams increase in sputum weight improve CFQ-R-RSS by 0.54 points.

### Lung function and HRQoL

**Fig 3:**
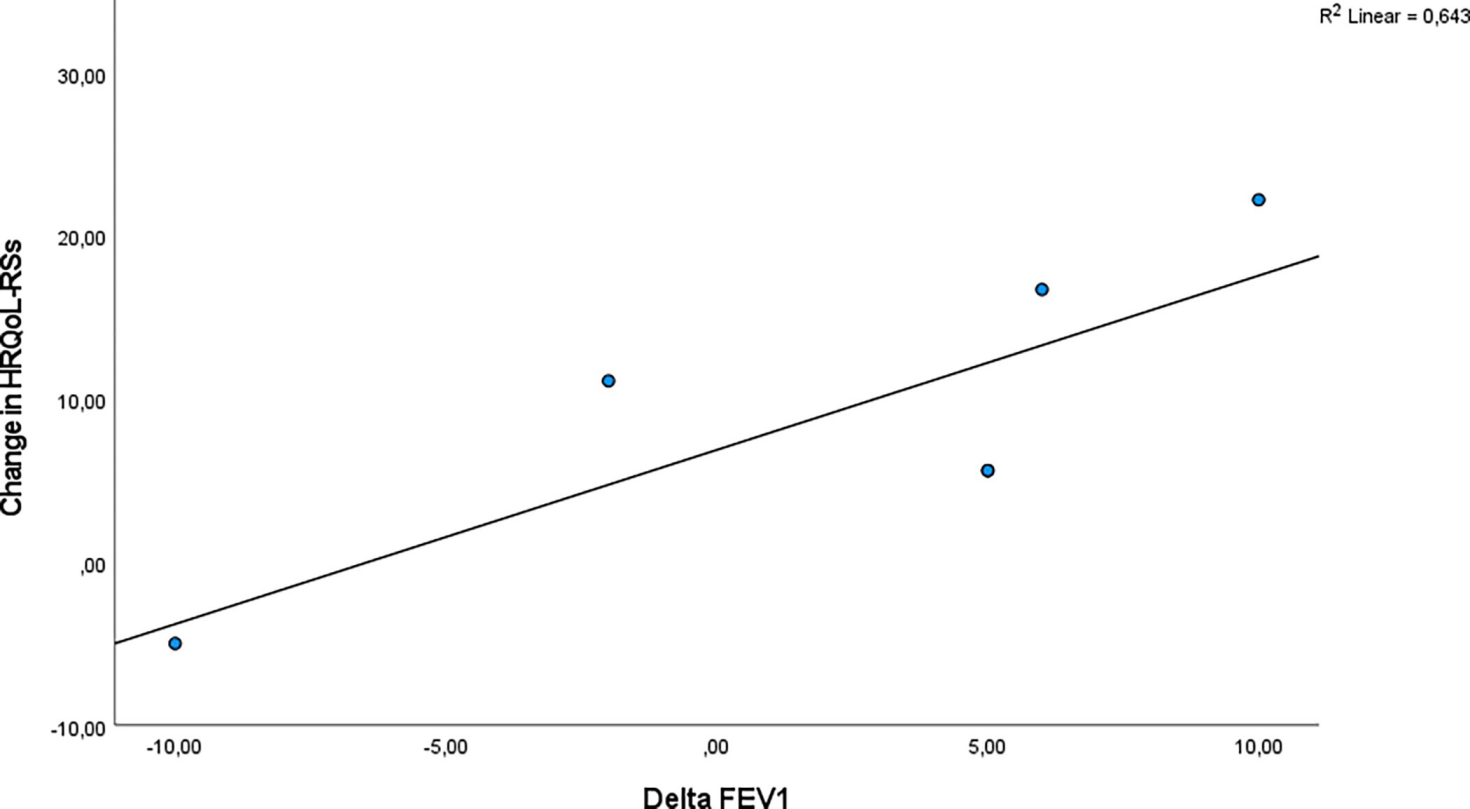
The correlation between change in FEV_1_ (start and end of study) and the change in CFQ-R-RSS (start and end of study).

There was a positive correlation for the relation between delta FEV_1_ and CFQ-R-RSS r = 0.82 (p = 0.04, R2 = 0.643). The correlation is presented in Fig 3.

**Fig 4:**
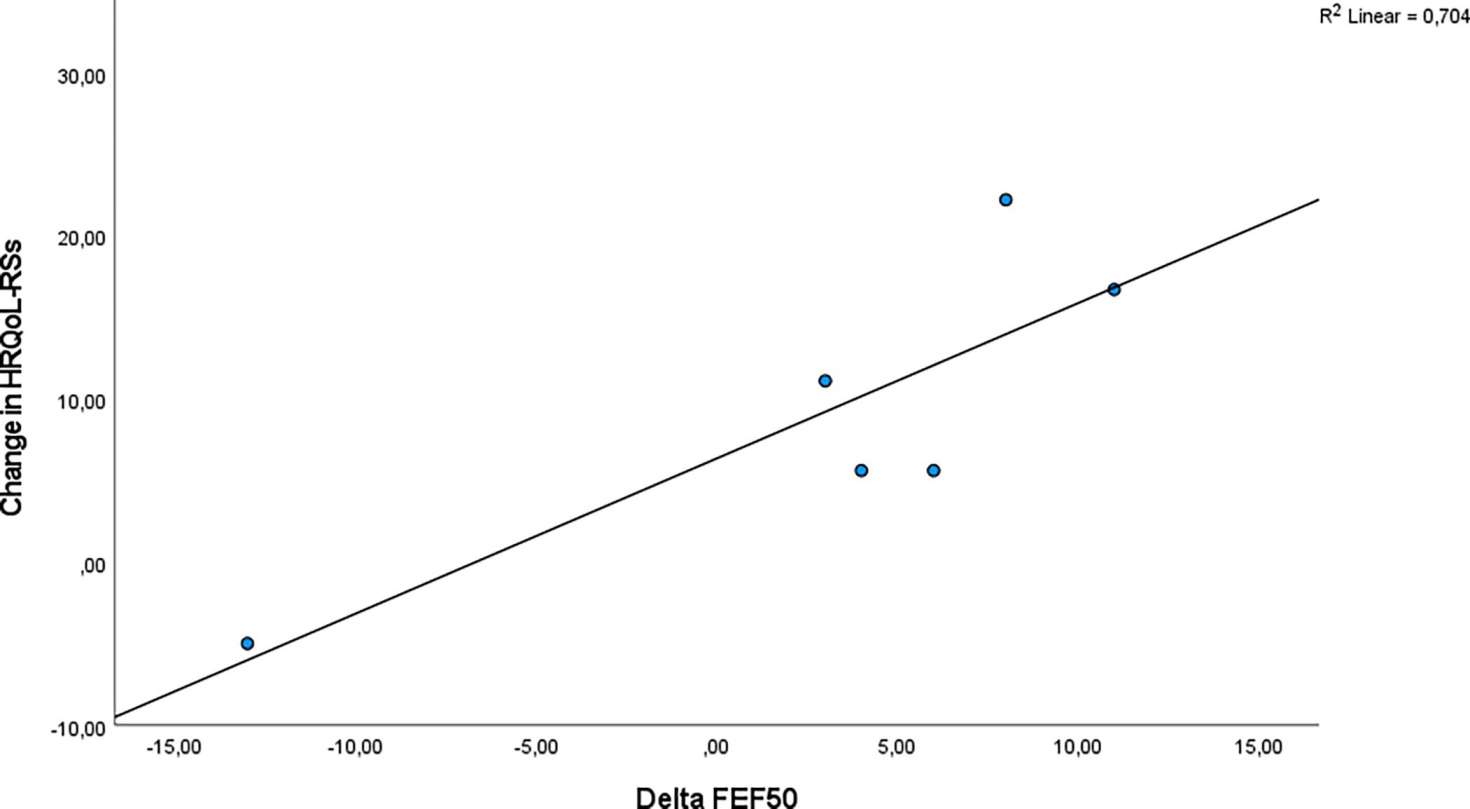
The correlation between change in FEF50 (start and end of study) and the change in CFQ-R-RSS (start and end of study).

There was also a positive correlation between delta FEF50 and CFQ-R-RSS r = 0.84 (p = 0.04, R2 = 0.704) (Fig 4).

There was no significant correlation between change in lung function and other CFQ-R domains.

We found in multiple linear regression analyses with backward elimination technique that delta FEF50 was more strongly associated with CFQ-R-RSS than delta FEV1.The relation between CFQ-R-RSS (dependent variable) and delta FEF50 in univariate linear regression analyses was Beta 0.95 (0.10, 0.40) (p = 0.037), implying that an increase in FEF50 of 1 percent predicted improves CFQR-RSS with 0.95 points. In a multiple linear regression model with both sputum weight and delta FEF50 in the analysis, only sputum weight remained significantly associated with CFQ-R-RSS.

## Discussion

CFQ-R respiratory symptoms scores in individuals with CF were improved after the airway clearance intervention period. We found a clinically important change in respiratory symptoms score in the majority of the participants, which clearly exceeded MID ≥ 4 p. This is an important finding with relevance to the airway clearance management of CF pulmonary disease. In addition, a positive change was found in the CFQ-R Health Perceptions in all participants. Further, a significant positive correlation was found between total sputum weight (g) produced by the participants during the study (week 1–8), and change in CFQ-R Respiratory Symptoms Scores, and a positive correlation was found between change in lung function and CFQ-R-Respiratory Symptoms Scores.

### Impact of Airway Clearance Physiotherapy on HRQoL

The CFQ-R was designed and validated for use as a clinical endpoint to evaluate the effect of interventions in CF. However, to our knowledge, few studies have demonstrated impact of ACP on HRQoL [7, 18].

In a recent overview of six Cochrane Reviews, the authors reported no definitive conclusions on the effectiveness and safety of ACTs in relation to HRQoL in CF [7]. Two reviews compared Positive Expiratory Pressure (PEP) with oscillating devices [24, 25], and included four long-term trials using different HRQoL scales. None reported any significant difference between the techniques for any of the HRQoL domains.

One long-term study by Pryor et al assessed five ACTs in adults with CF and found no difference in HRQoL as measured with Short Form-36 (SF-36) and Chronic Respiratory Questionnaire (CRQ) [26]. However, generic measures, such as the SF-36 lack sensitivity to change and the use of disease-specific measures are recommended [17] by both the US Food and Drug Administration and European Medicines Agency. A long-term study by McIlwaine et al assessed HRQoL comparing the efficacy of high-frequency chest wall oscillation with PEP over 1 year in 88 subjects with CF. They found a significant reduction in exacerbations in subjects using PEP. However, no significant difference in HRQoL scores was found between groups, as measured by CFQ-R, and the changes in Respiratory Symptoms scores within or between groups did not reach the MID [27].

In the present trial, the CFQ-R Respiratory Symptoms Scores were improved after the intervention. Clinically meaningful changes were documented in the majority of participants, with improvements clearly exceeding the MID. Thus, these results may reflect the experience of clinical response to treatment in individual participants. Participation in the study implied that participants removed more sputum than usual due to the intervention. Hence, the changes in HRQoL may be related to increased sputum removal, thereby also improving lung function parametres.

### HRQoL and the association with sputum weight

The main rationale for ACP in CF is to improve ventilation and deposition of inhaled medication and to enhance mucus clearance and expectoration of bronchial secretions when needed [8]. The ultimate goal in sputum producers is further to improve sputum removal in a gentle and effective way [8, 19].

However, while some previous studies have favoured one technique over another, other studies have not found significant differences in sputum production [4]. Pryor et al reported on a long-term comparative study of ACTs and found no statistically significant difference among the regimens. Sputum weight was criticized as lacking in validity as an endpoint for studies of ACTs [26]. Contradictory findings may be due to differences in study design, treatment protocols, methods, and outcome measures [28]. Recently, in an overview of six Cochrane reviews, authors reported no clear differences between ACTs in terms of sputum production, and a lack of information on which to draw conclusions [7].

To our knowledge, this is the first trial to assess HRQoL and the association with sputum clearance in CF. This trial found a significant, positive correlation between total sputum weight and the changes in CFQ-R Respiratory Symptoms Scores. The explanation for this finding may likely be due to the original trial design and the intervention as a whole addressing sputum removal, that is, techniques and treatment method, duration and frequency [8].

### HRQoL and the association with lung function

As ACP may improve lung function, FEV_1_% predicted is commonly used as outcome measure in clinical trials to assess the effectiveness of interventions [17]. FEV_1_ has been considered an appropriate surrogate outcome for clinical trials since it reflects disease status, and low lung function is strongly associated with decreased HRQOL [29].

Several studies have used pulmonary function measures to assess comparative effects in ACT studies [4]. However, the benefit of ACT on lung function is less clear [17]. Recently, Wilson et al found no differential effects between PEP-therapy versus oscillating devises in relation to lung function after six months of treatment [7]. Authors reported they were unable to draw any definite conclusions for all other comparisons linked to FEV_1_ and there were no clear differences in terms of FVC, FEF25-75 [7]. In a Cochrane review on nebulized hypertonic saline, hypertonic saline was found to be equally effective during as before ACT after a single treatment session. The timing had no substantial effect on the change in lung function [30].

The suitability and reliability of lung function as outcome measure for treatment efficacy has been questioned in three previous long-term studies [31]. The authors did not find lung function to be a sensitive measure when assessing change in the rate of pulmonary function decline [27, 31]. Other studies have demonstrated weak-moderate correlations between HRQoL and lung function [13]. However, the present study found a strong, positive correlation between change in lung function and changes in CFQ-R Respiratory Symptoms Scores. Thus, the association between HRQoL and lung function may reflect the effect of increased sputum removal related to the airway clearance intervention.

### HRQoL and the association with sputum and lung function

By addressing sputum removal, results demonstrated improvement in HRQoL and lung function. Not surprisingly, the benefit observed in respiratory symptoms reflects the higher amount of sputum that was expectorated during the intervention, which was associated with a benefit in lung function. It was anticipated a priori that increased sputum production might impact on lung function and HRQoL by way of improvement in respiratory symptoms.

Thus, the CFQ-R Respiratory Symptoms Scale was found to be a valid domain for this study and was sensitive to change.

It has been questioned whether participating in clinical trials influences perceptions of HRQoL due to increased attention and better treatment during the study period [27], which is true of any study. However, regression analysis from this study showed that sputum weight was more strongly associated with the CFQ-R Respiratory Symptoms Scale than lung function. Hence, it is likely that the main effects of the intervention were improved sputum clearance.

### Intervention

The present intervention used nebulised saline in horizontal positions during treatment alternating with either SCT or FET in random order [8]. The significant changes in respiratory symptoms and the improvement in lung function were found both in participants using hypertonic saline and isotonic saline.

To date, no clear evidence for the optimal duration and frequency of ACP exists [4, 18]. Previous ACT studies have typically performed interventions from 20–45 minutes duration twice daily [3, 27] and mostly in the sitting position. Length of time for treatment has also been individualized [26].

The present study used a treatment duration of approximately 60 minutes, twice a week over eight weeks. Thus, the benefits observed may reflect that longer treatment duration adds greater benefit due to increased sputum clearance. Hence, the clearance effect of the intervention may explain the positive changes seen in CFQ-R Respiratory Symptoms and lung function from their baseline values, and the associations found between HRQoL, sputum production and lung function.

### Relationship between clinical variables and measures of HRQoL

The relationship between clinical variables and measures of HRQoL can be illustrated by the conceptual model from Wilson and Cleary The model classifies different measures of health outcomes and can be used to identify measures, evaluate interventions, and improve quality of patient care [32]. The relationship is outlined on a continuum with causal associations and increasing complexity, in terms of biological and physiological factors, symptoms status, functioning, general health perceptions and overall quality of life. Relationships can be considered bidirectional [32].

**Fig 5:**
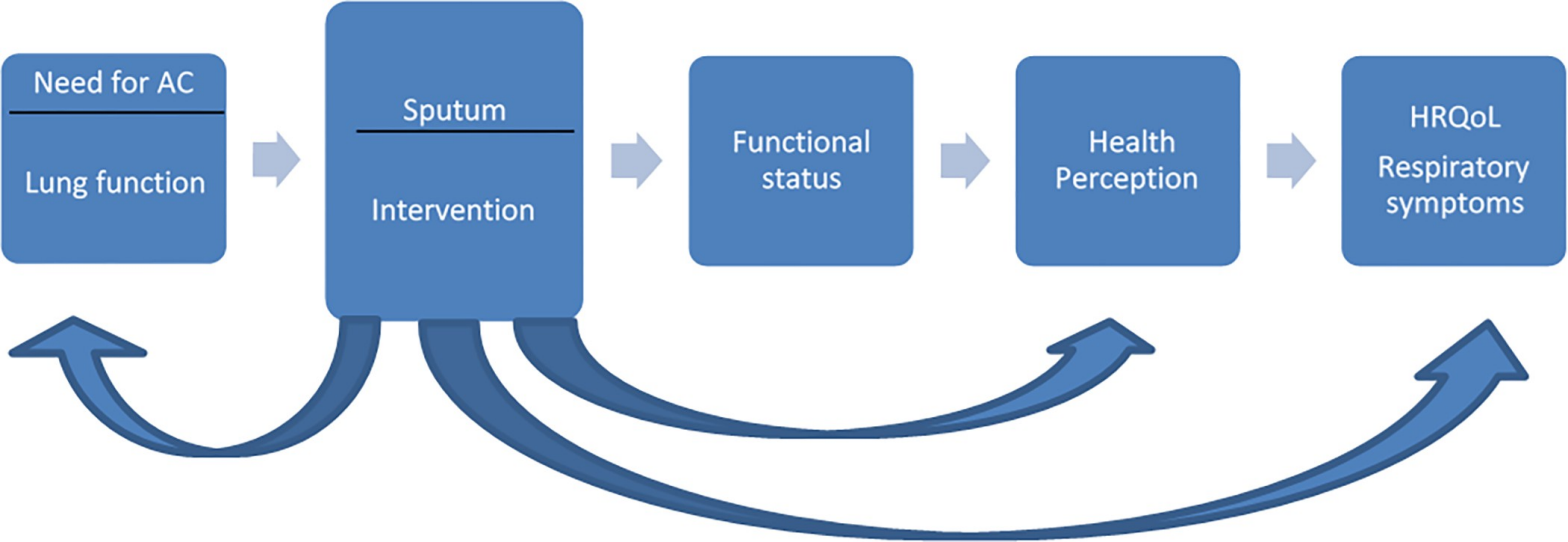
Need for airway clearance, study intervention and the relationships with HRQoL and lung function.

This study has demonstrated a linkage between different types of outcomes and a relationship between clinical variables and HRQoL (Fig 5).

By addressing the need for airway clearance in the intervention, we have demonstrated a clinically relevant change in CFQ-R-RSS and associations with sputum weight and lung function. Thus, sputum may be considered as a risk factor and a key factor, which can be modified in treatment strategies. By emphasizing sputum removal, respiratory symptoms improved, and so did health perceptions. In addition, improvement in respiratory symptoms was associated with critical clinical variables (lung function). Thus, a clinical intervention that reduces symptoms may also improve HRQoL in chronic diseases [11]. Hence, causal and bidirectional relationships have assisted in identifying sputum clearance as an important factor that affects symptoms and health perceptions, which can be modified by optimised and effective treatments, like the present working model and treatment method [20].

### Strengths and limitations

Long-term randomized controlled trials using clinical meaningful outcome measures have been suggested for future research [3, 18]. However, there may be poor adherence to a study protocol [33] and dropouts may be a challenge [31]. The need for alternative designs has been encouraged [31]. This study has demonstrated that increased sputum removal and airway clearance led to positive effects on self-reported HRQoL and lung function.

The strength of our study was the excellent adherence to the original trial design [8] with participants completing 16 interventions in total, with no dropouts. The results indicated that the participation of individuals in N-of-1 RCTs can influence the impact of an airway clearance intervention on HRQoL [8]. At the same time the perceived treatment burden scores were reduced or equal in 4 of 6 participants (S1 Table) and increased in 2. However, the intervention as a whole was well accepted, when considering the absence of dropouts.

Additional benefits may be enhanced skills and self-management, which in turn may influence adherence to maintenance treatment [8] and impact on health status. However, more studies are needed to determine the optimal duration and frequency of ACP interventions to derive the impact on HRQoL. A long-term trial with optimized intervention may further reveal information of importance to maintenance treatment and presuppositions for adherence.

Our results have also highlighted a relationship between sputum production and dimensions of HRQoL that are linked to other key health outcomes. Thus, this study has provided new research questions and insight into the effect of this new treatment method on HRQoL in CF, which may be of value in future ACP studies. The focus should be on target group and optimised intervention according to need, and outcome measures that are likely to change, such as the CFQ-R.

A limitation was the small sample size. However, this study was performed as part of a previously described pilot study of a series of N-of-1 randomised controlled trials [8]. Comparisons with other studies are difficult due to variability in interventions, duration and frequency, methodologies (design, subjects, outcomes, data analysis), and outcome measures.

The clinical implication of our study is that the intervention as a whole has demonstrated clinically meaningful changes in the CFQ-R respiratory symptoms domain in the majority of the participants. The strong and positive association between primary and secondary outcome measures highlights the relationships between symptoms, clinical variables and HRQoL. Further, the role of sputum clearance as an intervening variable that likely mediate the effects should be explored both in clinical settings and in future studies.

It remains important to establish individualized and optimized maintenance treatment, ensure performance skills and involve patients in shared-decision making. Importantly, surveillance and follow-up are essential to adjust treatment and treatment goals to avoid growing treatment complexity. Individual issues and needs demand different interventions. In the era of CFTR-modulators, this is especially important. Hence, the treatment in less sputum producers might address optimized ventilation and drug deposition more than airway clearance.

## Conclusions

Participation in the present study led to improvement in HRQoL and MID ≥ 4 in the CFQ-R Respiratory Symptoms domain in the majority of participants. Based on this finding, we conclude our results to be of clinical relevance. Further, a significant correlation was found between total sputum weight (g) and the positive changes in CFQ-R Respiratory Symptoms Scores, and between the changes in lung function and the CFQ-R Respiratory Symptoms Scores. These findings warrant further investigation with regard to ACP and its content, duration and frequency. A long-term trial adapted to the target group with appropriate outcome measures may further reveal information of importance to maintenance treatment. More studies are needed to investigate ACP and ACTs and possible beneficial effects on HRQoL, and to explore the use of HRQoL in clinical trials.

## Supporting information

S1 Table HRQoL results. Details data on CFQ-R domains, as measured at baseline (week 1) and at the end of study (week 8).
(PDF)
