# Supplementary material for: Airway clearance physiotherapy and health-related quality of life in cystic fibrosis
Source: PLoS One. 2022 Oct 18;17(10):e0276310. doi: 10.1371/journal.pone.0276310 (PMC9578613; doi:10.1371/journal.pone.0276310)
Supplement: S1 Table — Details data on CFQ-R domains, as measured at baseline (week 1) and at the end of study (week 8). (PDF) [file pone.0276310.s001.pdf]

Health-related quality of life (HRQoL) results.

HRQoL measured with CFQ-R at baseline (week1) and at the end of study (week 8).

**Participant**	1		2		3		4		5		6	
**Week**	1	8	1	8	1	8	1	8	1	8	1	8
**Variables**	**Scores**											
Physical functioning	100	95.8	66.6	58.3	83.3	83.3	91.6	29.2*	83.3	87.5	79.2	75.0
Role functioning	91.6	91.6	91.6	83.3	58.3	75.0	75.0	91.6	75.0	75.0	100	100
Vitality	41.6	75.0	66.6	75.0	41.6	50.0	50.5	58.3	33.3	25.0	50.0	50.0
Emotion	81.6	53.3	100	100	80.0	80.0	93.3	93.3	40.0	26.6	93.3	80.0
Social functioning	81.1	77.7	83.3	77.8	66.6	55.5	77.7	72.2	33.3	33.3	77.7	72.2
Body image	66.6	55.5	100	100	100	100	77.7	66.6	55.5	44.4	100	88.8
Eating	77.7	55.5	100	100	100	100	100	100	88.8	88.8	77.7	77.7
Treatment burden	66.6	55.5	77.7	66.6	44.4	77.7	44.4	55.5	55.5	55.5	55.5	77.7
Health perception	55.5	66.6	66.6	88.9	44.4	66.6	44.4	55.5	33.3	44.4	44.4	66.6
		+ 11.1		+ 22.3		+ 22.2		+ 11.1		+11.1		+ 22.2
Weight	33.3	66.6	66.6	100	100	100	66.6	66.6	100	100	66.6	100
Respiratory symptoms	55.5	77.7	55.5	66.6	44.4	50.0	55.5	61.1	66.6	61.6	55.5	72.2
		+ 22.2		+ 11.1		+ 5.6		+ 5.6		- 5.0		+16.7
Nutrition	77.7	66.6	100	100	44.4	66.6	77.7	77.7	55.5	44.4	77.7	100

*Participant 4: Reduced score on the physical functioning variable due to other illness in treatment pair/week 7.
